# Structural design principles that underlie the multi-specific interactions of Gα_q_ with dissimilar partners

**DOI:** 10.1038/s41598-019-43395-0

**Published:** 2019-05-03

**Authors:** Shir Navot, Mickey Kosloff

**Affiliations:** The Department of Human Biology, Faculty of Natural Science, University of Haifa, Haifa, 3498838 Israel

**Keywords:** Computational biology and bioinformatics, GTP-binding protein regulators

## Abstract

Gα_q_ is a ubiquitous molecular switch that activates the effectors phospholipase-C-β3 (PLC-β3) and Rho guanine-nucleotide exchange factors. Gα_q_ is inactivated by regulators of G protein signaling proteins, as well as by PLC-β3. Gα_q_ further interacts with G protein-coupled receptor kinase 2 (GRK2), although the functional role of this interaction is debated. While X-ray structures of Gα_q_ bound to representatives of these partners have revealed details of their interactions, the mechanistic basis for differential Gα_q_ interactions with multiple partners (i.e., Gα_q_ multi-specificity) has not been elucidated at the individual residue resolution. Here, we map the structural determinants of Gα_q_ multi-specificity using structure-based energy calculations. We delineate regions that specifically interact with GTPase Activating Proteins (GAPs) and residues that exclusively contribute to effector interactions, showing that only the Gα_q_ “Switch II” region interacts with all partners. Our analysis further suggests that Gα_q_-GRK2 interactions are consistent with GRK2 functioning as an effector, rather than a GAP. Our multi-specificity analysis pinpoints Gα_q_ residues that uniquely contribute to interactions with particular partners, enabling precise manipulation of these cascades. As such, we dissect the molecular basis of Gα_q_ function as a central signaling hub, which can be used to target Gα_q_-mediated signaling in therapeutic interventions.

## Introduction

Gα_q_ is a ubiquitous protein hub that interacts with multiple protein partners as part of its function as a molecular switch. Gα_q_ belongs to the family of heterotrimeric (αβγ) G proteins that are activated by G protein-coupled receptors (GPCRs), promoting the exchange of GDP for GTP in the active site of the Gα subunit^1^. Activated Gα_q_ dissociates from the βγ subunits and stimulates two major classes of downstream effectors – phospholipase C-β (PLC-β) isozymes^2^ and Rho guanine nucleotide exchange factors (RhoGEFs), such as p63RhoGEF^3^. These Gα_q_ effectors can regulate cellular processes such as smooth muscle contraction, platelet activation, immune responses, and neuronal function^4–12^, and are crucial in pathologies such as cancer, neurological disorders, and cardiovascular diseases^13–17^.

The ability of proteins such as Gα subunits to interact with multiple partners, often using partially overlapping interfaces, is termed multi-specificity^18^. Indeed, the interactions of Gα_q_ with diverse partners (i.e., GPCRs, regulators of G protein signaling (RGS) proteins, phospholipase C-β isoforms, G protein-coupled receptor kinases (GRKs), and RhoGEFs) define Gα_q_ as a multi-specific protein^19^. Gα_q_, as well as Gα subunits of the homologous G_i_ sub-family, can be inactivated by RGS proteins. The latter bind Gα-GTP and allosterically accelerate the intrinsic GTPase activity of the Gα subunit, thereby acting as GTPase-activating proteins (GAPs)^20,21^. Uniquely, the PLC-β effector also shows GAP activity towards Gα_q_^22–26^, although the significance of this dual regulation is unclear^27,28^. In another Gα-specific interaction, proteins containing “GoLoco motifs” can bind specifically to members of the G_i_ sub-family^29,30^, functioning as guanine nucleotide dissociation inhibitors (GDIs). Gα_q_ can also interact with GRKs, and in particular, with GRK2^31,32^. The central function of GRK2 is to mediate initial steps in GPCR desensitization^33,34^, although it was also proposed to sequester activated Gα_q_ from other effectors, and was suggested to function as a GAP towards Gα_q_^31,35,36^. Overall, the residue-level determinants of Gα_q_ interactions with its multiple partners remain to be explicitly defined, and in particular using a common and objective framework for analyzing interactions with different partners.

Gα_q_ can interact with its partners via different regions of the Gα subunit. However, the residue-level determinants of Gα_q_ multi-specificity are still not sufficiently understood. Complexes of Gα_q_ with representatives of these partners have been solved, namely Gα_q_-RGS2^37^, Gα_q_-RGS8^38^, Gα_q_-PLC-β3^39^, Gα_q_-GRK2^40^, and Gα_q_-p63RhoGEF^41^. Gα_q_ contains two structural domains, the GTPase domain, which is also found in other G proteins, and the α-helical domain, which is unique to Gα subunits. The GTPase domain contains three flexible regions called “switch regions” (Sw I, II, and III), which undergo conformational changes, depending on whether the Gα subunit binds GTP or GDP^42^. These regions contain two residues that are critical for the GTPase reaction – a catalytic arginine in Sw I (Gα_q_-Arg183) and a catalytic glutamine in Sw II (Gα_q_-Gln209). In addition, Gα GTPase domains also include an “effector-binding site” that was previously defined as the C-terminal half of Sw II, the α3 helix, and the subsequent loop that connects the latter to the β5 strand^43^. This region was shown to participate in the binding of Gα subunits to effectors, such as the binding of Gα_s_ to adenylyl cyclase^43^. RGS proteins were shown to substantially interact with the Gα GTPase domain, yet were also shown to interact with the Gα helical domain^37,38,44–46^. More recently, it was suggested that the helical domain is a major determinant of the specific interactions between Gα_q_ and RGS2^47^. PLC-β3 was shown by Waldo *et al*. to engage three Gα_q_ regions in the GTPase domain, Sw I, Sw II and the effector-binding site, while interactions with the Gα_q_ helical domain were not mentioned^39^. This study also suggested that a helix-turn-helix motif at the C-terminus of the PLC-β3 C2 domain determines its binding to Gα_q_ as an effector. On the other hand, a loop that connects EF hands 3 and 4 in PLC-β3 was shown to mediate its GAP function^24,39^. Similar to PLC-β3, p63RhoGEF binds the Gα_q_ effector-binding site via a conserved helix-turn-helix motif^19,39,41^, but also binds the C-terminal region of Gα_q_^41^. Diversely, while GRK2 also binds to the Gα_q_ effector-binding site, this partner lacks a helix-turn-helix motif and, despite sharing an RGS homology (RH) domain with RGS proteins, GRK2 and RGS proteins were suggested to bind to non-overlapping surfaces of Gα_q_^40^. Indeed, a precise and quantitative definition of which Gα_q_ residues contribute to the interface with each partner is lacking, as well as a clear-cut delineation of where these interfaces overlap.

Previous mutagenesis studies tested a limited number of Gα_q_ residues, located in the switch regions and the effector-binding site, and showed them to be important for interactions with particular partners. Two residues in Gα_q_ Sw II, three residues in Sw III, and three residues in the effector-binding site were identified as playing roles in PLC-β activation^39,48^. Shankaranarayanan *et al*. identified two residues in Gα_q_ Sw III, one residue in the α3 helix, and one residue in the Gα_q_ C-terminal region (Tyr356) as important only for activating p63RhoGEF^49^. Site-directed mutagenesis also assigned four Gα_q_ residues in the GTPase domain as being important for binding GRK2 – one residue in Sw I, two residues in Sw III and one residue adjacent to Sw III^50^. This study also showed that mutations in four Gα_q_ helical domain residues impaired GRK2 binding. Finally, Tesmer *et* *al*. showed that four Gα_q_ residues in the Gα_q_ effector-binding site were also required for GRK2 binding^40^. Nevertheless, which Gα_q_ residues interact with all partners and which residues interact specifically with only one partner has yet to be defined.

Here, we used structural comparisons and energy-based calculations to produce a comprehensive map of the residue-level determinants of Gα_q_ multi-specificity. We used structure-based Finite Difference Poisson–Boltzmann (FDPB) and burial-based energy calculations to accurately pinpoint which amino acids contribute to the interaction of Gα_q_ with each of its different partners. We further identified unique Gα_q_ regions that specifically interact with GAPs and disparate Gα_q_ regions that interact with effectors. We also identified Gα_q_ regions that contribute to interactions with multiple partners, and particular Gα_q_ residues that specifically contribute to interactions with only one select partner.

## Results

### Delineation of structurally-similar domains and sub-structures in Gα_q_ partners

**Figure 1:**
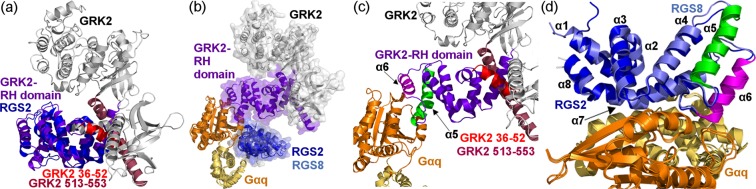
GRK2 and RGS2/8 contain a structurally similar RH domain that binds to non-overlapping surfaces of Gα_q_ via different sub-structures of the RH domain. (**a**) Superimposition of the structures of RGS2 and GRK2 (from PDB IDs: 4EKD and 2BCJ, respectively). The structurally-aligned core of the RGS2 and GRK2 RH domains are colored blue and purple, respectively. Two helices in the N- and C-terminal regions of the GRK2 RH domain that do not have equivalent sub-structures in RGS2 (GRK2 residues 36–52 and 513–553) are colored red and maroon, respectively, while the unaligned parts of GRK2 are colored gray. (**b**) Superimposition of the Gα_q_-RGS2, Gα_q_-RGS8 and Gα_q_-GRK2 complexes (PDB IDs: 4EKD, 5DO9 and 2BCJ, respectively), using only the coordinates of Gα_q_. Gα_q_ subunits are visualized as ribbon diagrams, colored orange (GTPase domain) and yellow (helical domain). GRK2 and RGS2/8 are colored as in panel a and the three Gα_q_ partners are also visualized as transparent molecular surfaces. (**c**) The binding pose of the GRK2 RH domain relative to the GTPase domain of Gα_q_, shown as in panel b, with GRK2 helices α5 and α6 colored green and magenta, respectively. (**d**) The binding pose of the RGS2 and RGS8 RH domains, relative to Gα_q_, shown as in panel b but rotated 90° about the X-axis. RGS helices are numbered, with the RGS2 and RGS8 α5 and α6 helices colored green and magenta, as the corresponding GRK2 helices in panel c.

Towards analyzing the multi-specificity determinants of Gα_q_ with its partners at the individual residue level, we first characterized and precisely defined the structural building blocks used by Gα_q_ partners to recognize Gα_q_ using structural alignments – to compare the available experimentally-solved complexes of Gα_q_ with GRK2, RGS2, RGS8, PLC-β3, and p63RhoGEF^37–41,51^. The structure-based ECOD classification database classifies GRK2 residues 29–185 and RGS2 residues 69–200 as homologous RGS homology domains (RH domains) belonging to the same structural family (i.e., the same ECOD F-group). On the other hand, Lodowski *et al*. defined the GRK2 RH domain as two discontinuous segments, the first being a nine-helix bundle (residues 30–185), and the second corresponding to an extended helix (residues 513–547)^52^. Structural alignment of GRK2 and RGS2 showed that the cores of the RH domains in RGS2 and GRK2 are similar. Specifically, GRK2 residues 52–176 and RGS2 residues 81–200 structurally aligned with a root mean square deviation (RMSD) of 2.8 Å (Fig. 1a). In contrast, a helical segment in the N-terminal region of the previously defined GRK2 RH domain (residues 36–52) and the extended helix at the C-terminus of this domain (residues 513–553) have no structural equivalents in RGS2 (Fig. 1a). Aligning the complexes using only the coordinates of Gα_q_ showed that the RH domains of RGS2 and GRK2 indeed interact with distinct regions of Gα_q_ (Fig. 1b). Furthermore, as noted previously^52^, we observed that the GRK2 and the RGS2/8 RH domains use different regions to interact with Gα_q_. In GRK2, the region encompassing helices α5 and α6 binds Gα_q_ (Fig. 1c), while in RGS2 and RGS8 these helices are peripheral to the interface and helix α7 is an RGS-unique determent of the interaction (Fig. 1d). Taken together, these results show that only the core RH domain of GRK2 (residues 52–176) is homologous to the RGS domains of RGS2 and RGS8 and is relevant to a comparison of interactions with Gα_q_. Moreover, because of their disparate binding poses, dissimilar Gα_q_ residues are expected to contribute to the binding of RGS proteins and GRK2.

**Figure 2:**
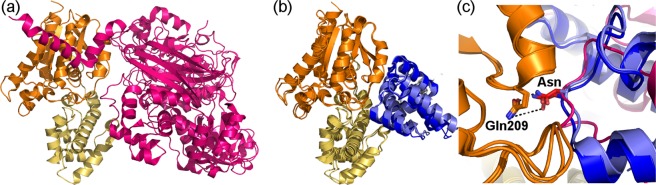
PLC-β3 and RGS domains are structurally dissimilar, except for one asparagine residue in both partners that interacts similarly with Gα_q_. (**a**) The complex of Gα_q_ with PLC-β3 (PDB ID: 3OHM). Gα_q_ is shown as in Fig. 1b, PLC-β3 is shown as a magenta ribbon diagram. (**b**) Superimposition of the complexes of Gα_q_ with RGS2 and RGS8 (PDB IDs: 4EKD and 5DO9, respectively). Gα_q_ is shown as in panel a, RGS2 and RGS8 are shown as blue and light blue ribbon diagrams, respectively. (**c**) Superimposition of the complexes of Gα_q_ with PLC-β3, RGS2 and RGS8, using only the coordinates of Gα_q_ for the superimposition. One asparagine residue (“Asn”, namely Asn260/149/122 in PLC-β3/RGS2/RGS8) adopts the same orientation towards Gα_q_ in all of these structures and interacts with Gα_q_ similarly in all structures - in particular with the Gα_q_ catalytic residue Gln209 (shown in sticks, with hydrogen bonds shown as dashed black lines).

We next compared the structures of Gα_q_ with PLC-β3, RGS2, and RGS8^37–39^, noting that their interfaces with Gα_q_ are indeed structurally dissimilar (Fig. 2a,b). On the other hand, in all three of these interfaces, an asparagine residue (Asn260 in PLC-β3, Asn149 in RGS2 and Asn122 in RGS8) adopts the same orientation (Fig. 2c), interacting with the catalytic glutamine that is essential for GTP hydrolysis, as previously observed^39^.

**Figure 3:**
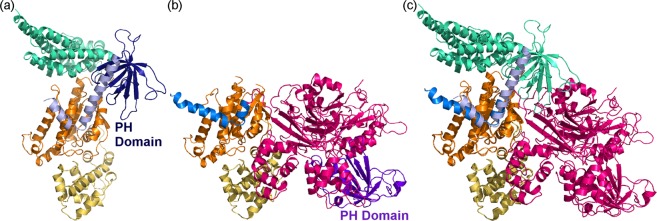
PLC-β3 and p63RhoGEF bind Gα_q_ with a structurally-similar helix-turn-helix motif. (**a**) The structure of Gα_q_–p63RhoGEF (PDB ID: 2RGN). p63RhoGEF is shown as a ribbon diagram, with its Dbl homology (DH) domain colored turquoise, its Pleckstrin homology (PH) domain colored dark blue, and its helix-turn-helix motif (residues 468–490) colored light blue. (**b**) The structure of Gα_q_–PLC-β3 (PDB ID: 3OHM). PLC-β3 is shown as a magenta ribbon diagram, except for the PH domain that is colored purple. The PLC-β3 helix-turn-helix motif (residues 852–874) is colored blue. (**c**) Superimposition of the complexes of Gα_q_ with PLC-β3 and p63RhoGEF, using only the coordinates of Gα_q_ for the superimposition. p63RhoGEF is colored turquoise and PLC-β3 is colored magenta, with the helix-turn-helix motifs colored light blue and blue, as in panels a and b, respectively. Gα_q_ in all panels is shown as in Fig. 1b.

Finally, we compared the structures of Gα_q_ with p63RhoGEF and PLC-β3^39,41^. Both of these proteins contain a structurally-similar pleckstrin homology (PH) domain. According to the ECOD database, p63RhoGEF residues 343–490 and PLC-β3 residues 12–146 adopt a PH domain-like fold. While this domain is structurally similar in both proteins, it interacts directly with Gα_q_ in p63RhoGEF, whereas in PLC-β3, this domain is far from the interface with Gα_q_ (Fig. 3a cf. b). On the other hand, a shorter helix-turn-helix motif in both p63RhoGEF and PLC-β3 binds Gα_q_ similarly (Fig. 3c). These structurally-aligned helix-turn-helix motifs include residues 468–490 in p63RhoGEF and residues 852–874 in PLC-β3, binding Gα_q_ at its previously-defined^43^ effector-binding site (Fig. 3c).

Our analyses suggest that because of the structural dissimilarities between Gα_q_ partners and the dissimilarities in their binding poses in relation to Gα_q_, an alternative approach to precisely delineate the multi-specificity determinants of Gα_q_ should be used. This approach involves assessing which residues are common and which are unique to such interactions by focusing on the Gα_q_ side of the interface and by analyzing which Gα_q_ residues contribute to each interaction using a quantitative energy-based approach.

### Residue-level mapping of Gα_q_ interactions with individual partners

**Figure 4:**
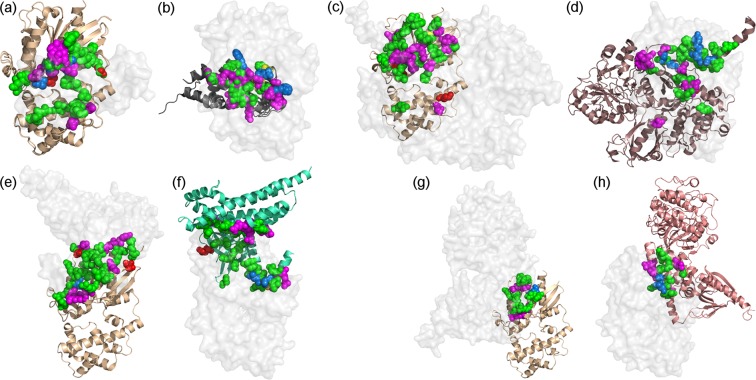
Residues that contribute substantially to interactions in Gα_q_ complexes with RGS2/8, PLC-β3, p63RhoGEF, and GRK2. (**a**) Gα_q_ residues that substantially contribute to interactions with RGS2 and RGS8. The Gα_q_-RGS2/8 crystal structures (PDB IDs: 4EKD and 5DO9) were superimposed using the Gα_q_ coordinates. (**b**) RGS2 and RGS8 residues that substantially contribute to interactions with Gα_q_. The crystal structures of Gα_q_-RGS2 (PDB ID: 4EKD) and Gα_q_-RGS8 (PDB ID: 5DO9) were superimposed using RGS coordinates, shown as gray ribbons. (**c**) Gα_q_ residues that contribute substantially to interactions with PLC-β3 (PDB ID: 3OHM). (**d**) PLC-β3 (maroon ribbon) residues that contribute substantially to interactions with Gα_q_. **(e**) Gα_q_ residues that contribute substantially to interactions with p63RhoGEF (PDB ID: 2RGN). (**f**) p63RhoGEF (cyan ribbon) residues that contribute substantially to interactions with Gα_q_. (**g**) Gα_q_ residues that contribute substantially to interactions with GRK2 (PDB ID: 2BCJ). (**h**) GRK2 (pink ribbon) residues that contribute substantially to interactions with Gα_q_. In all panels, residues that contribute substantially to interactions with the cognate partner are shown as spheres and colored according to the type of energy contribution: side-chain polar/electrostatic and non-polar contributions, magenta; side-chain polar/electrostatic contribution only, red; main-chain polar/electrostatic contribution only, yellow; main-chain polar/electrostatic and non-polar contributions, blue; non-polar contributions only, green. In panels a,c,e, and g – Gα_q_ is shown as a gold ribbon and the cognate partner as a transparent gray molecular surface. In panels b,d,f, and h – Gα_q_ is shown as a transparent gray molecular surface.

To map the individual residues that contribute to the interactions of Gα_q_ with each of its partners, we characterized the five complexes detailed above using an energy-based computational methodology developed previously by our lab^45–47,53–55^. The FDPB method was used to calculate the net electrostatic and polar contributions (ΔΔG_elec_) of each residue within 15 Å of the Gα_q_-partner interface in each complex. For each residue, we separately calculated the electrostatic contributions from the side chain and/or those originating from the main chain of each residue. Residues that substantially contribute to the interaction were defined as those contributing ΔΔG_elec_ ≥ 1 kcal/mol to the interactions (i.e. twice the numerical error of the electrostatic calculations)^56^. Note that this approach calculates the net difference between the interaction of a residue with its protein partner in relation to its interaction with the water and ions in the solvent, and thereby identifies only residues that are calculated to substantially contribute to binding. Non-polar energy contributions (ΔΔG_np_) were calculated as a surface-area proportional term by multiplying the per-residue surface area buried upon complex formation by a surface tension constant of 0.05 kcal/mol/Å^2^. Residues with substantial non-polar contributions were defined as those contributing ΔΔG_np_ ≥ 0.5 kcal/mol to the interactions (namely, more than 10 Å^2^ of each protein surface is buried upon complex formation). To reduce false positives and negatives, we applied a consensus approach across comparable biological replicates in multiple PDB structures or across multiple dimers in an asymmetric unit (see Methods and Supplementary Figs S1 and S2), which substantially improved the accuracy of our predictions. Residues thus calculated to contribute substantially to intermolecular interactions were mapped to the structure of each individual protein (Fig. 4).

Our results show that in all of the complexes analyzed, the majority of Gα_q_ residues contribute to interactions with the cognate partners via non-polar interactions (Fig. 4a,c,e,g, Supplementary Fig. S3, Supplementary Tables 1–4). A similar majority of non-polar contributing residues was also observed in PLC-β3 (Fig. 4d), p63RhoGEF (Fig. 4f), and GRK2 (Fig. 4h). In contrast, the majority of RGS residues that contribute to interactions with Gα_q_ do so via electrostatic contributions (Fig. 4b). The electrostatic dominance in interactions of RGS domains with Gα subunits was also observed in interactions of Gα_o_ and Gα_i_ with various RGS domains^46^. The number of Gα_q_ residues that contribute to interactions with RGS2 and RGS8 are 27 and 25, respectively. Complexes with PLC-β3 and p63RhoGEF involve a larger number of Gα_q_ residues, namely 36 and 31 residues, respectively. In contrast, in the complex with GRK2, only 16 Gα_q_ residues contribute to the interaction. While about a quarter of the Gα_q_ residues contributing to interactions with RGS domains are located in the Gα_q_ helical domain, only three Gα_q_ helical domain residues contribute to interactions with PLC-β3, and no contributions with p63RhoGEF and GRK2 originate from the Gα_q_ helical domain.

On the opposing face of these interfaces, the structurally similar helix-turn-helix motifs in PLC-β3 and p63RhoGEF (Fig. 3) contain 12 residues that contribute to interactions with Gα_q_; four of these residues are identical and contribute similarly to interactions with Gα_q_ in p63RhoGEF and in PLC-β3 (Supplementary Fig. S4). As mentioned above (Fig. 2c), Asn260 in PLC-β3 and the corresponding Asn149/122 in RGS2/8 adopt essentially the same orientation and interact similarly with the catalytic Gα_q_ Gln209 residue. Our calculations predict that this residue contributes to interaction with Gα_q_ via side-chain electrostatic and non-polar interactions in all three structures.

### Comparison of the multi-specific interactions of Gα_q_ with its different partners

**Figure 5:**
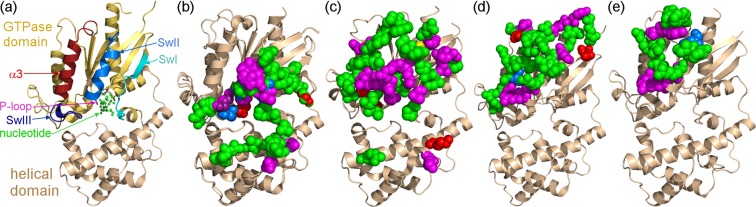
Comparison of Gα_q_ residues contributing to interactions with different partners. (**a**) Structural regions in Gα_q_ that can interact with its partners. Gα_q_ is shown as a ribbon diagram colored light orange (GTPase domain) and gold (helical domain). The α3 helix and the subsequent loop are colored maroon. The P-loop is colored magenta and the three switch regions are marked as follows: Sw I, teal; Sw II, blue; and Sw III, purple. The nucleotide is shown as balls and sticks, colored green. **(b**) Gα_q_ residues that substantially contribute to the interaction with RGS2 and RGS8. (**c**) Gα_q_ residues that substantially contribute to the interaction with PLC-β3. (**d**) Gα_q_ residues that substantially contribute to the interaction with p63RhoGEF. (**e**) Gα_q_ residues that substantially contribute to the interaction with GRK2. Gα_q_ structures (as in the complexes analyzed in Fig. 4) are depicted as gold ribbon diagrams, with partner structures omitted for clarity. Gα_q_ residues that substantially contribute to interactions with each partner are shown as spheres and colored according to the type of energy contribution, as in Fig. 4.

To precisely define the shared and unique determinants responsible for interactions of Gα_q_ with its partners, we compared which Gα_q_ residues contribute to the interaction with each partner (Fig. 5). We thus identified a single residue in the Gα_q_ P-loop that contributes to interactions with RGS proteins, and one or two residues in the Gα_q_ β1 strand, which immediately precedes the P-loop, that contribute to interactions with PLC-β3 and p63RhoGEF. As mentioned above, the Gα_q_ helical domain makes no contributions to interactions with p63RhoGEF or GRK2. Rather, this domain mostly interacts with RGS domains, and in a limited fashion with PLC-β3 (Fig. 5b,c cf. d,e). Residues in the Gα_q_ Sw II contribute to interactions with all partners, while Sw I and III residues only contribute to interactions with RGS domains and PLC-β3 (Fig. 5). Between nine and 12 Gα_q_ residues in the region immediately following Sw III (residues 248–265, termed here the α3 motif, Fig. 5a) only contribute to interactions with PLC-β3, p63RhoGEF and GRK2 (Fig. 5c–e). Several Gα_q_ residues closer to the C-terminus (residues 319–321 and 353–357) only contribute to the interaction with p63RhoGEF (Fig. 5d).

**Figure 6:**
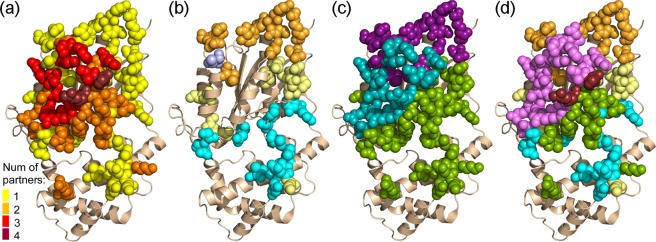
Multi-specificity analysis of Gα_q_. (**a**) Gα_q_ residues that substantially contribute to interactions with its partners, classified according to the number of binding partners interacting with each residue, colored as in the key. Gα_q_ is shown as a gold ribbon. The contributions of RGS2 and RGS8 residues were combined into a consensus map representing both RGS proteins. (**b**) Gα_q_ residues that uniquely contribute to interactions with only one partner (i.e. those marked with yellow spheres in panel a), shown as spheres and colored according to the identity of the partner with which they interact: p63RhoGEF, orange; PLC-β3, yellow; RGS2/8, cyan; GRK2, light blue. (**c**) Gα_q_ residues that interact uniquely with GAPs (PLC-β3/RGS proteins) as opposed to non-GAPs (p63RhoGEF/GRK2). Gα_q_ residues that contribute to interactions with these partners are shown as spheres and colored as follows: residues that contribute to interactions with p63RhoGEF and/or GRK2 (contributions to “effectors” only) are colored purple, residues that contribute to interactions with PLC-β3 and/or RGS2/8 (contributions to “GAPs” only) are colored green, and residues that contribute to interactions with both effectors and GAPs are colored teal. (**d**) Gα_q_ residues that interact with particular effector combinations. Contributing Gα_q_ residues are shown as spheres and colored as follows: residues that contribute to interactions with all three effectors (p63RhoGEF, GRK2, and PLC-β3) are colored lilac, residues that contribute to interactions with PLC-β3 and with RGS2/8 are colored green, residues that contribute to interactions with PLC-β3 only are colored yellow, residues that contribute to interactions with only p63RhoGEF are colored orange, residues that contribute to interactions with only RGS2/8 are colored cyan and residues that contribute to interactions with all four partners are colored maroon.

The majority of Gα_q_ residues contribute to interactions with only one partner (Fig. 6a,b). Most residues in Sw II and some residues in Sw III contribute to interactions with both effectors and GAPs (Fig. 6c), while the helical domain and Sw I contribute to interactions only with GAP proteins (Fig. 6c,d). Moreover, the Gα_q_ helical domain contains five residues that contribute to interactions with RGS proteins alone (Fig. 6b,d). Residues in the Gα_q_ effector-binding site do not contribute to interactions with RGS proteins, and true to the name of this site, contribute only to interactions with effectors (Fig. 6d). Overall, there are only two Gα_q_ residues, located in Sw II, that contribute to interactions with all four partners (Fig. 6a,d).

**Figure 7:**
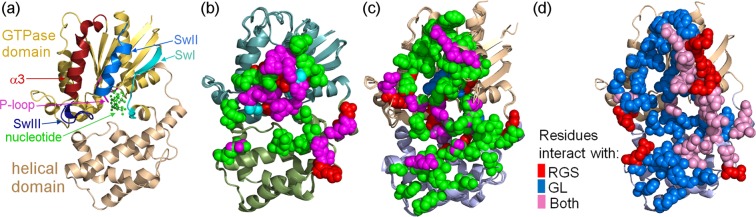
Multi-specificity analysis of Gα_i_ residues contributing to interactions with RGSs and GoLoco motifs. (**a**) Structural regions in Gα_i_ that can interact with its partners. Gα_i_ is shown as a ribbon diagram colored light orange (GTPase domain) and gold (helical domain). The α3 helix and subsequent loop are colored maroon. The P-loop is colored magenta and the switch (Sw) regions are colored as follows: Sw I, teal; Sw II, blue; and Sw III, purple. The nucleotide is shown as balls and sticks, colored green. (**b**) Gα_i1_ residues that substantially contribute to interactions with high-activity RGS domains, shown as spheres and colored according to the type of energy contribution, as in Fig. 4. The following three crystal structures of Gα_i1_–RGS complexes (with PDB IDs) were superimposed: Gα_i1_–RGS1 (PDB ID: 2GTP), Gα_i1_–RGS4 (PDB ID: 1AGR), and Gα_i1_–RGS16 (PDB ID: 2IK8). Gα_i_ subunits are shown as ribbon diagrams, colored according to Gα domains: teal (GTPase domain) and olive (helical domain). (**c**) Gα_i1_ residues that substantially contribute to interactions with GoLoco (GL) motifs, shown as spheres and colored as in panel b. The following three crystal structures of Gα_i1_–GoLoco complexes (with PDB IDs) were superimposed: Gα_i1_–GL-RGS14 (PDB ID: 2OM2), Gα_i1_–GL-LGN4 (PDB ID: 4G5Q), and Gα_i3_–GL-LGN3 (PDB ID: 4G5S). Gα_i_ is visualized as a ribbon diagram, colored gold (GTPase domain) and light blue (helical domain). (**d**) Interactions of Gα_i_ with GoLoco motifs versus RGS domains. Gα_i_ residues that substantially contribute to the interactions are shown as spheres, colored according to the key. Gα_i_ is visualized as a ribbon diagram, colored as in panel c.

To gain a wider perspective on the Gα family in terms of multi-specific interactions with different partners, we compared the interactions of Gα_q_ analyzed above with the interactions of Gα_i_ with RGS proteins and GoLoco motifs (Fig. 7). Between 25 and 28 Gα_i_ residues contribute to interactions with different RGS proteins (Fig. 7b), while 35–41 Gα_i_ residues contribute to interactions with the GoLoco motifs in RGS14 and LGN (Fig. 7c). Similar to Gα_q_, the majority of Gα_i_ residues contributing to interactions with RGS proteins rely on electrostatic interactions (Fig. 7b), with Gα_i_ regions interacting with RGS proteins being similar to Gα_q_ regions that engage RGS proteins (Figs 5b cf. 7b). In contrast, the majority of Gα_i_ interactions with the GoLoco motifs involve non-polar interactions (Fig. 7c). Unlike the interactions of RGS domains with either Gα_i_ or Gα_q_, we found six to eight residues in the Gα_i_ P-loop that contribute to interactions with GoLoco motifs. A third of Gα_i_ residues contributing to interactions with the RGS14 GoLoco motif are located in the helical domain, while a sixth of the residues contributing to the interaction of Gα_i_ with the GoLoco motif of LGN are located in the helical domain (Supplementary Fig. S5). Moreover, the majority of the contributing residues in the Gα_i_ helical domain are involved only in interactions with GoLoco motifs (Fig. 7d). On the other hand, the Gα_i_ Sw I and Sw II regions contain numerous residues that contribute to interactions with either the GoLoco motifs or with RGS proteins, while Gα_i_ Sw III makes only limited contributions to interactions with either the GoLoco motifs or the RGS proteins (Fig. 7b,c). Lastly, the Gα_i_ α3 motif contains eight residues that contribute only to interactions with the GoLoco motifs but not with RGS proteins.

## Discussion

Our energy-based computational methodology provides a quantitative framework to compare the multi-specific interactions of Gα_q_ with RGS2/8, PLC-β3, p63RhoGEF, and GRK2 at the individual residue level. Our results revealed that only residues from one Gα_q_ region, Sw II, interact with all of its partners, GAPs and effectors alike – suggesting that Sw II is a necessary and central motif for the recognition of the activated state of Gα_q_ by its partners, rather than a major specificity determinant towards a specific partner. The Gα_q_ effector-binding site is also rather promiscuous, interacting with all partners except for RGS proteins, while the Gα_q_ helical domain, Sw I and parts of Sw III only interact with RGS proteins and PLC-β3. Our multi-specificity analysis identified numerous residues across the surface of Gα_q_ that interact with only one partner, such as the Gα_q_ C-terminal region that exclusively interacts with p63RhoGEF and several helical domain residues that only interact with RGS domains. This analysis enables precise manipulation of individual interactions. Our results also show that most of the Gα_q_ effector-binding site interacts only with effectors, as opposed to the Gα_q_ regions that uniquely interact with GAPs. In particular, the Gα_q_ effector-binding site contains eight residues that contributed to interactions with all three effectors – PLC-β3, p63RhoGEF and GRK2; four Gα_q_ positions contribute to interactions with two effectors. The effector-binding site also contains two residues that contributed specifically to interactions only with PLC-β3, three residues that contributed to interactions only with p63RhoGEF, and one residue that contributed to interactions only with GRK2. Therefore, the Gα_q_ effector-binding site also includes specificity determinants for particular Gα_q_ effectors.

Our calculations also delineate which interactions with particular Gα_q_ regions can underlie GAP activity. The Gα_q_ Sw I region contains residues that contribute to interactions only with PLC-β3 and RGS proteins, suggesting a functional role for Sw I interactions with proteins possessing GAP activity towards Gα_q_. This hypothesis is sustained by a previous study^57^ that used FTIR spectroscopy to show that RGS4 interactions with the intrinsic arginine finger of Gα_i_, which is located in Sw I, are important for GAP activity. We also found that the Gα_q_ helical domain contains seven residues that contribute to interactions with RGS proteins. We note that, in addition to RGS proteins, the Gα_q_ helical domain also contributes to interactions with PLC-β3 – interactions that were not discussed or investigated in previous studies. This suggests that interactions with the Gα_q_ helical domain play a common role in mediating the function of these different GAPs. On the other hand, we observed no interactions between residues in the Gα_q_ effector-binding site and RGS proteins. Relevantly, the extended loop in PLC-β3, which connects EF hands 3 and 4 and was shown to be a critical component for GAP activity^24,39^, interacts mostly with the Gα_q_ Sw I and the N-terminus of Sw II. Taken together, this suggests that the Gα_q_ effector-binding site interacts solely with effectors or with regions responsible for effector activation and does not play a role in interactions with GAPs. On the other hand, no residues in Gα_q_ Sw I or in the Gα_q_ helical domain contribute to interactions with GRK2. Although GRK2 contains an RH domain that is similar to RGS domains in RGS proteins, all GRK2 interactions with Gα_q_ are with Sw II and the effector-binding site. Therefore, while Carman *et al*. suggested that GRK2 has weak GAP activity towards Gα_q_^31^, our results did not identify any interactions that might underlie a GAP activity of GRK2. Overall, our results suggest that the Gαq effector-binding site interacts solely with downstream effectors, while the Gα_q_ helical domain and Sw I region interact uniquely with GAPs.

Our analysis of Gα_q_ multi-specificity also pinpointed specific determinants responsible for particular partner interactions. Several residues in the Gα_q_ helical domain consist of a unique specificity determinant towards RGS proteins, while only one residue in this domain is specific to PLC-β3. We found eight residues in the Gα_q_ C-terminal region (319–321 and 353–357) that contribute solely to interactions with p63RhoGEF. The role of residues in this Gα_q_ region in mediating interactions with p63RhoGEF is supported by a previous study that mutated Tyr356 in the Gα_q_ C-terminal region and showed that p63RhoGEF binding was impaired^49^. However, our results suggest the Gα_q_ C-terminal region that contributes to specific interactions with p63RhoGEF is more extensive than previously suggested. Furthermore, the Gα_q_ N-terminal region preceding the P-loop also contains two residues that contribute to interactions only with p63RhoGEF, while one residue in this region contributes only to the interaction with PLC-β3. Most of these contributing residues were not investigated previously and represent new Gα_q_ specificity determinants towards these partners.

Finally, we compared the interactions of Gα_i_ and Gα_q_ with multiple partners and showed that interactions with RGS proteins involving the switch regions were nearly identical, while RGS interactions with the helical domain differed substantially. Our analysis showed that RGS proteins contributed essentially the same to interactions with the Sw I and Sw III regions in both Gα_q_ and Gα_i_, suggesting these regions do not play a role in determining RGS domain specificity towards Gα_q_ and Gα_i_. This contrasts with a previous study that suggested, based on visual inspection of the crystal structure, that the Gα Sw I and Sw III regions contain key residues responsible for the selectivity of RGS domains for Gα_q_ and Gα_i_^38^. Relevantly, both GoLoco motifs and RGS domains interacted with Sw I in the Gα subunits. Taken together, these commonalities suggest that proteins whose function involves the guanine nucleotide, possessing either GAP or GDI activity, bind Sw I as part of their function. On the other hand, the extensive interactions of GoLoco motifs with the Gα_i_ helical domain, combined with their interactions with the effector-binding site, are unique to the Gα_i_ sub-family and stand out from the interactions of Gα_q_ with its partners. More general conclusions regarding the multi-specificity determinants of the entire Gα family will require applying the approach used here to additional members.

In summary, the energy-based computational analysis described here presents a precise comparison of Gα_q_ interactions with multiple partners using a common quantitative framework. This framework allows extension of such analyses to other Gα subunits involved in interactions with different partners and to additional multi-specific proteins. Our analysis suggests that multiple Gα_q_ residues contribute to the discrimination between different protein partners, and provides a structural basis for precisely mutating Gα_q_ residues in order to manipulate and unravel its interactions *in vivo* and in cells. From a wider perspective, our results provide residue-level insight into protein-protein interactions that drive cellular signaling processes and lay the basis for specifically targeting Gα_q_-mediated signaling in therapeutic interventions.

## Methods

### Protein structures

The following representative 3D structures were used in our analysis and visualization of Gα subunits with different classes of partners (PDB IDs are provided for each structure): Gα_q_–RGS2 (4EKD)^37^, Gα_q_–RGS8 (5DO9)^38^, Gα_q_–PLC-β3 (3OHM, 4QJ3, 4QJ4, 4QJ5)^39,51^, Gα_q_–p63RhoGEF (2RGN)^41^, Gα_q_–GRK2 (2BCJ)^40^, Gα_i1_–RGS4 (1AGR)^58^, Gα_i1_–RGS16 (2IK8)^44^, Gα_i1_–RGS1 (2GTP)^44^, Gα_i1_–RGS14-GoLoco (2OM2, 3ONW, 3QI2)^59–61^, Gα_i1_–LGN-GoLoco4 (4G5Q)^62^, Gα_i3_–LGN-GoLoco3 (4G5S)^62^, and Gα_i3_–LGN-GoLoco4 (4G5R, 4G5O)^62^. Missing short segments in PDB entries 3OHM, 4QJ3, 4QJ4 and 4QJ5 (PLC-β3 residues 90–98 and 472–574), entry 2RGN (p63RhoGEF residues 367–373 and 396–403), entry 2BCJ (GRK2 residues 476–491 and 668–689), entry 3QI2 (RGS14-GL residues 511–512), and entry 4G5S (Gα_i3_ chain A residue 117 and chain B residues 204–205) were modeled using Loopy^63^ and partial or missing side chains were modeled using Scap^63^. Hydrogen atoms were added using CHARMM, and the structures were subjected to conjugate gradient minimization with a harmonic restraint force of 50 kcal/mol/Å^2^ applied to the heavy atoms. Structure alignments were performed using the Combinatorial Extension (CE) method, as implemented in the RCSB protein comparison tool (https://www.rcsb.org/pdb/workbench/workbench.do). 3D structural visualization was carried out with the PyMol molecular graphics program (https://www.pymol.org/).

### Energy calculations to map residue-level specificity determinates

We followed the methodology described previously^45–47,53–55,64^ to analyze the per-residue contributions of Gα_q_ residues to interactions with their partners (PLC-β3, p63RhoGEF, RGS2/8, GRK2) in the crystal structures mentioned above. The Finite Difference Poisson–Boltzmann (FDPB) method, as implemented in DelPhi^65^, was used to calculate the net electrostatic and polar contributions (ΔΔG_elec_) of each residue found within 15 Å of the dimer interface. For each residue, electrostatic contributions from the side chain and/or originating from the main chain were calculated separately. Residues contributing ΔΔG_elec_ ≥ 1 kcal/mol to the interactions (twice the numerical error of the electrostatic calculations) were deemed as substantially contributing to the interaction^45,56^. Non-polar energy contributions (ΔΔG_np_) were calculated as a surface-area proportional term by multiplying the per-residue surface area buried upon complex formation, calculated using surfv^66^, by a surface tension constant of 0.05 kcal/mol/Å^2 ^^56^. Residues contributing ΔΔG_np_ ≥ 0.5 kcal/mol to the interactions (namely, those that bury more than 10 Å^2^ of each protein surface upon complex formation) were defined as making substantial non-polar contributions^64^. To reduce false positives and negatives, we applied a consensus approach across comparable biological replicates in multiple PDB structures (3OHM, 4QJ3, 4QJ4, 4QJ5 – see Supplementary Fig. S1; 2OM2, 3ONW, 3QI2; 4G5R, 4G5O) or across multiple dimers in an asymmetric unit (5DO9 – see Supplementary Fig. S2; 2RGN; 4G5Q; 4G5S; 4G5R, 4G5O), substantially improving prediction accuracy.

## Supplementary information

Supplementary Information

## Data Availability

The datasets generated during or analyzed during the current study are available from the corresponding author on reasonable request.
