# Supplementary material for: Structural design principles that underlie the multi-specific interactions of Gαq with dissimilar partners
Source: Sci Rep. 2019 May 3;9:6898. doi: 10.1038/s41598-019-43395-0 (PMC6499889; doi:10.1038/s41598-019-43395-0)
Supplement: Supplementary file 1 — Supplementary Information [file 41598_2019_43395_MOESM1_ESM.pdf]

Supplementary Information

Structural design principles that underlie the multi-specific interactions of $G\alpha_q$ with dissimilar partners

Shir Navot[1] and Mickey Kosloff[1,*]

[1]The Department of Human Biology, Faculty of Natural Science, University of Haifa, Haifa, 3498838, Israel.

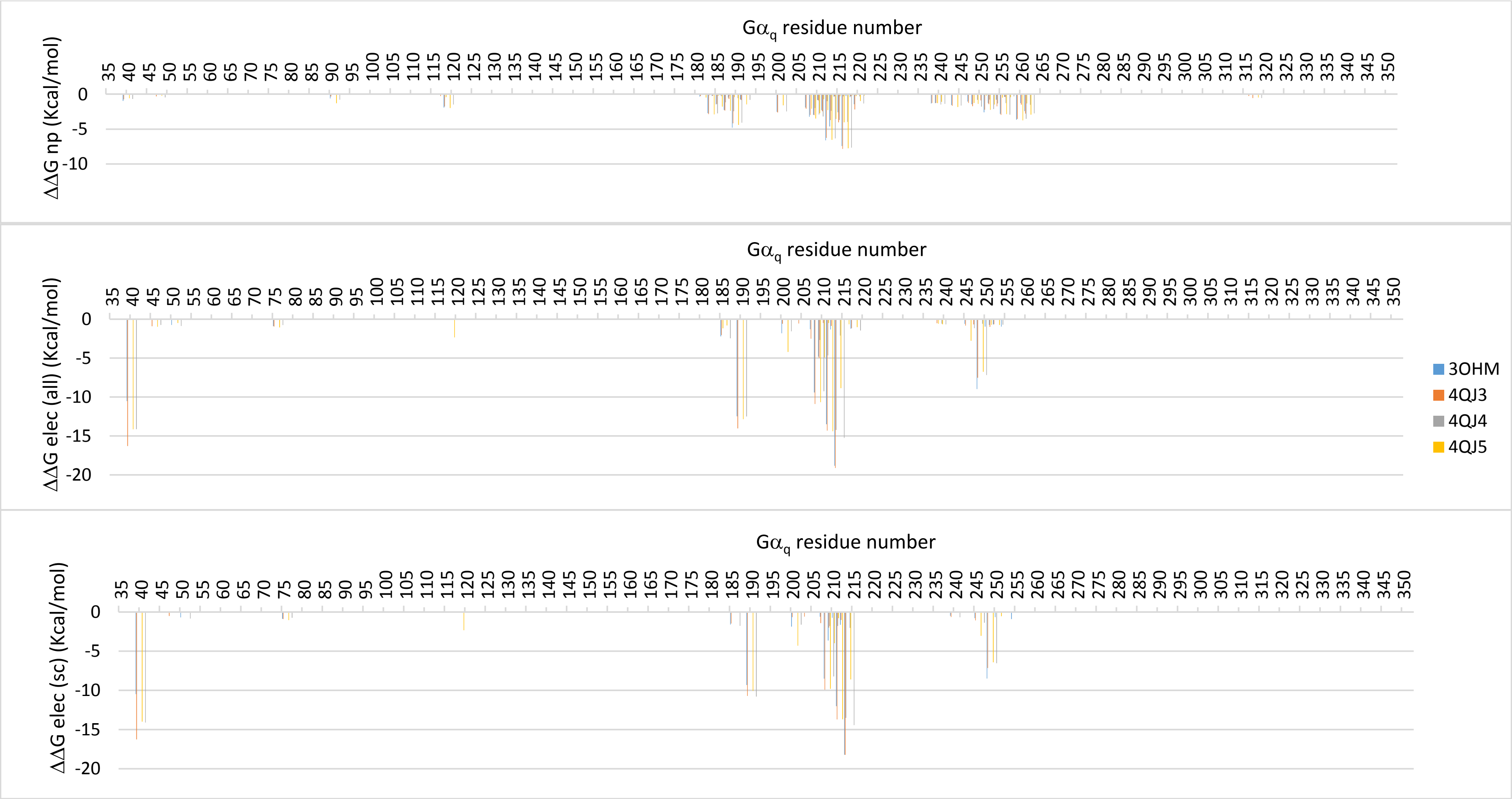

Supplementary Figure S1: Per-residue energy contributions to interactions of Gα$_q$ with PLC-β3 in four different X-ray structures. Panels show the results for non-polar (np) contributions, electrostatic contributions from entire residues (all), and electrostatic contributions from side-chains (sc), calculated as described in Methods for PDB IDs 3OHM, 4QJ3, 4QJ4, 4QJ5.

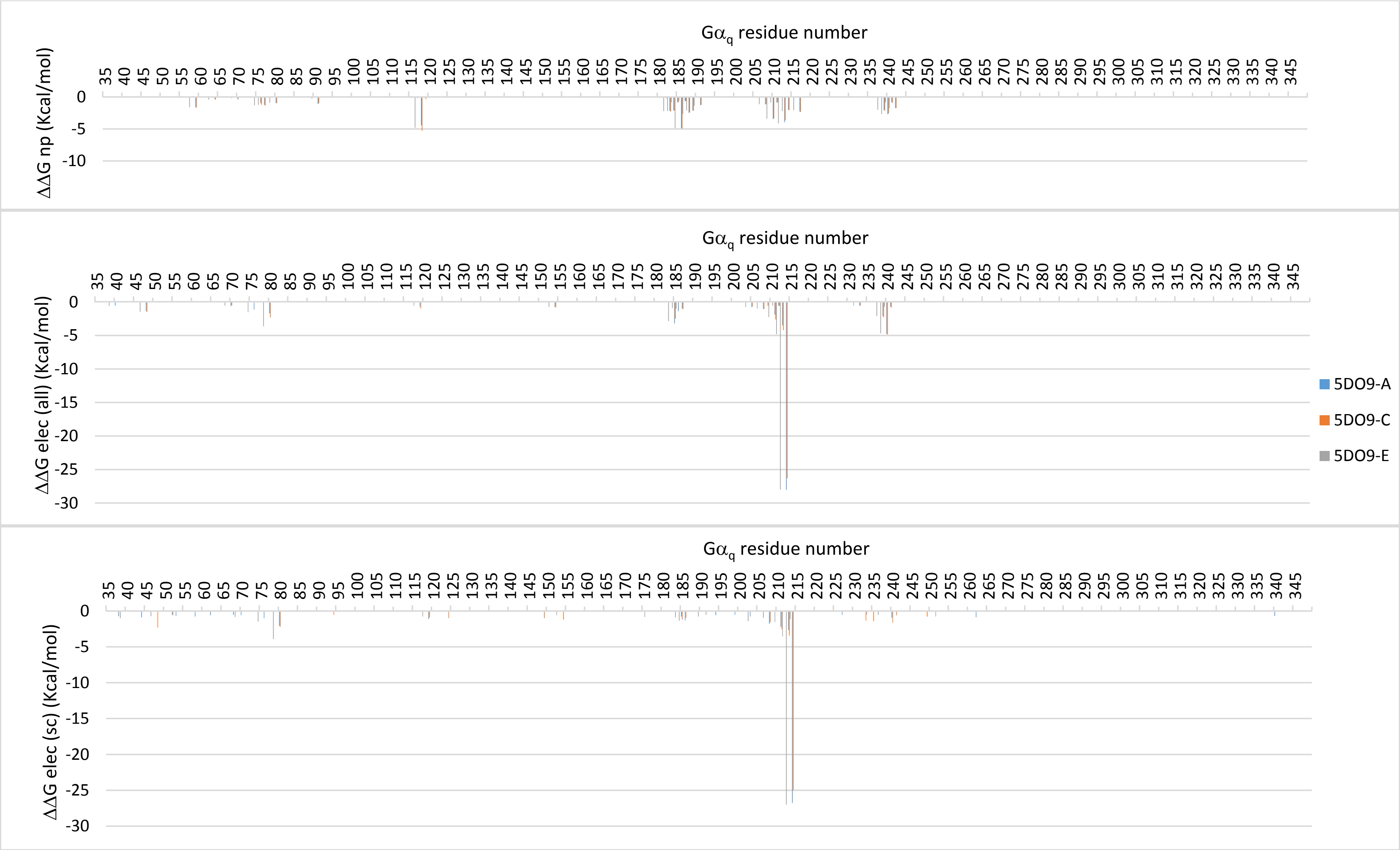

Supplementary Figure S2: Per-residue energy contributions to the interactions of Gα$_q$ with RGS8 in the three replicates in the asymmetric unit of PDB ID 5DO9. Panels show the results of the energy calculations for the interactions of Gα$_q$ (chains A, C, E) with RGS8 (chains B, D, F), as in Supplementary Fig. S1.

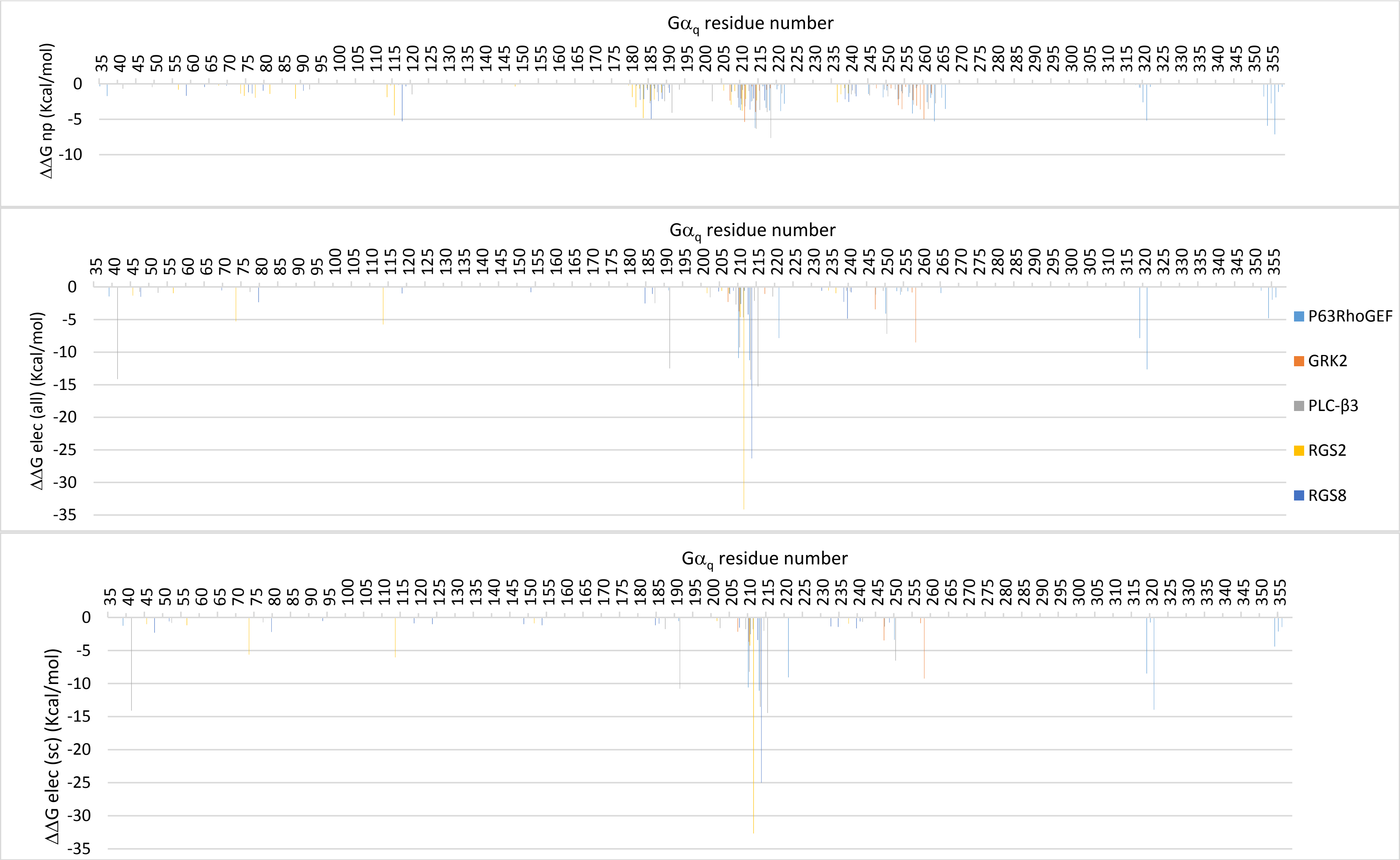

Supplementary Figure S3: Per-residue energy contributions to the interactions of Gα$_q$ with p63RhoGEF, GRK2, PLC-β3, RGS2, and RGS8. Panels show the results of the energy calculations for the interactions of Gα$_q$ with each partner (as detailed in Fig. 4), presented as in Supplementary Fig. S1.

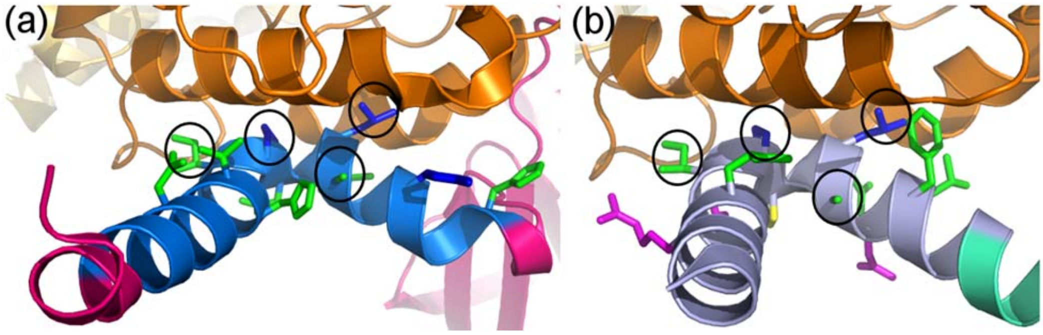

Supplementary Figure S4: The helix-turn-helix motifs in p63RhoGEF and PLC-β3 contain a core of four identical residues that interact similarly with Gα_q. (a) The helix-turn-helix region (blue ribbon) in PLC-β3 (pink ribbon), which aligns to p63RhoGEF. Residues predicted to contribute to interactions with Gα_q are shown as sticks and colored according to their energy contribution, as in Fig. 4. Gα_q is shown as a ribbon diagram colored orange. **(b)** The comparable helix-turn-helix region (light blue ribbon) in p63RhoGEF (teal ribbon). Gα_q and residues predicted to contribute to interactions with Gα_q are shown as in A. Residues that are identical in both proteins are marked with black circles (p63RhoGEF residues Ala474, Leu475, Pro478, and Ile479; PLC-β3 residues Ala858, Leu859, Pro862, and Ile863).

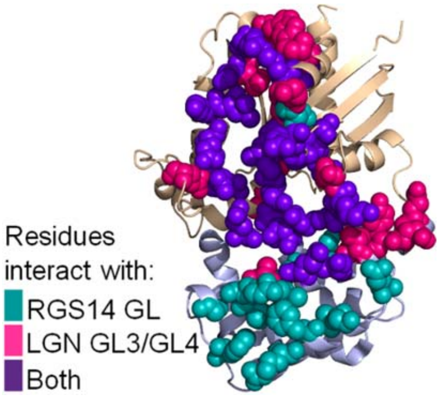

Supplementary Figure S5: Multi-specificity analysis of Gα_i residues contributing to interactions with RGS14-GoLoco and LGN-GoLoco motifs. Gα_i residues that substantially contribute to interactions are shown as spheres, colored according to the key. Gα_i is visualized as a ribbon diagram, colored gold (GTPase domain) and light blue (helical domain).

Supplementary Table 1: Gα_q per-residue energy contributions to interactions with PLC-β3.

Gα_q residue	Energy contributions to interactions with PLC-β3 [a]
K41	np + sc elec
K77	sc elec
R92	np
K120	np + sc elec
P185	np
T187	np + sc elec
I189	np
I190	np
E191	np + sc elec
P193	np
R202	np + sc elec
Q209	np + sc elec
R210	np + sc elec
S211	np + sc elec
E212	np
R213	np + sc elec
R214	np + sc elec
K215	np + sc elec
I217	np
H218	np
C219	mc elec
E221	np
V240	np
E241	np
E245	np
R247	sc elec
E249	np
E250	np + sc elec
K252	np
A253	np
L254	np
R256	np + sc elec
T257	np
Y261	np
P262	np
W263	np

[a] Energy contributions, calculated as described in Methods: np, non-polar; sc elec, side-chain electrostatic contribution; mc elec, main chain electrostatic contribution.

Supplementary Table 2: Gα_q per-residue energy contributions to interactions with RGS domains.

Gα_q residue	Energy contributions to interactions with RGS domains [a]
E49	np + sc elec
R60	np (RGS8) / sc elec (RGS2)
K77	np + sc elec
L78	np
Q81	sc elec (RGS8) / np (RGS2)
R92	np
K117	np + sc elec (RGS2)
E119	np
V184	np
P185	np
T186	np + mc elec
T187	np + sc elec
G188	np
I189	np
I190	np
Y192	np
Q209	np + sc elec
S211	np (RGS8) / np + mc elec (RGS2)
E212	np + sc elec
R214	np + sc elec
K215	np + sc elec
H218	np
V240	np + mc elec
E241	np + sc elec
S242	np
D243	np

[a] Energy contributions, calculated as described in Methods: np, non-polar; sc elec, side-chain electrostatic contribution; mc elec, main chain electrostatic contribution.

Supplementary Table 3: Gα_q per-residue energy contributions to interactions with p63RhoGEF.

Gα_q residue	Energy contributions to interactions with p63RhoGEF [a]
R37	np
E39	sc elec
R210	np + sc elec
R213	np + sc elec
R214	np
K215	np + sc elec
I217	np
H218	np
F220	np
E221	np + sc elec
N222	np
E245	np
E249	np
E250	np + sc elec
A253	np
L254	np + mc elec
R256	np
T257	np
I258	np
Y261	np
P262	np
W263	np
Q265	np
N266	np
D319	sc elec
S320	np
D321	np + sc elec
L353	np
K354	np + sc elec
E355	np + sc elec
Y356	np + sc elec
N357	np

[a] Energy contributions, calculated as described in Methods: np, non-polar; sc elec, side-chain electrostatic contribution; mc elec, main chain electrostatic contribution.

Supplementary Table 4: Gα_q per-residue energy contributions to interactions with GRK2.

Gα_q residue	Energy contributions to interactions with GRK2 [a]
R210	np + sc elec
R213	np + sc elec
R214	np
I217	np
H218	np
F220	np + mc elec
E221	np
E250	np + sc elec
L254	np
R256	np
T257	np
T260	np + sc elec
Y261	np + sc elec
P262	np
W263	np
Q265	np

[a] Energy contributions, calculated as described in Methods: np, non-polar; sc elec, side-chain electrostatic contribution; mc elec, main chain electrostatic contribution.

Supplementary Table 5: Gα_i per-residue energy contributions to interactions with RGS domains.

Gα_{i1} residue	Energy contributions to interactions with RGS domains[a]
E43	sc elec
E65	sc elec
Q68	np + sc elec
A71	np (RGS16)
V72	np (RGS16, RGS4)
S75	np
Q79	np (RGS16, RGS1)
R90	np + sc elec (RGS16) / np (RGS4, RGS1)
E115	sc elec
E116	np + sc elec
V179	np
K180	np + sc elec
T181	np + mc elec
T182	np + sc elec
G183	np + mc elec
I184	np
V185	np
D200	sc elec
Q204	np + sc elec
R205	np
S206	np
E207	sc elec
R208	sc elec (RGS16)
K209	np + sc elec
K210	np + sc elec
H213	np
A235	np + mc elec
E236	np + sc elec
E238	np

[a] Energy contributions, calculated as described in Methods: np, non-polar; sc elec, side-chain electrostatic contribution; mc elec, main chain electrostatic contribution.

Supplementary Table 6: Gα_i per-residue energy contributions to interactions with GoLoco (GL) motifs.

Gα_i residue	Energy contributions to interactions with GoLoco (GL) motifs [a]
L39	np (LGN-GL4, LGN-GL3)
G40	np + mc elec
A41	np + mc elec
G42	np
E43	np + mc elec
S44	sc elec (LGN-GL3)
K46	sc elec (RGS14-GL) / np (LGN-GL3)
S47	np
E65	sc elec (LGN-GL3)
Q68	np (LGN-GL3)
Y69	np (LGN-GL4, LGN-GL3)
V72	np (LGN-GL4, LGN-GL3)
S75	np (RGS14-GL, LGN-GL3)
N76	sc elec (RGS14-GL)
Q79	np + sc elec (RGS14-GL, LGN-GL4) / np (LGN-GL3)
S80	sc elec (RGS14-GL)
I82	np (RGS14-GL)
A83	np (LGN-GL3)
I85	np (RGS14-GL)
R86	np + sc elec (RGS14-GL)
G89	np (RGS14-GL)
F95	np (RGS14-GL)
R105	np (RGS14-GL)
F108	np (RGS14-GL)
A111	np (RGS14-GL)
G112	np (RGS14-GL)
E116	np (RGS14-GL)
Q147	np
N149	np + sc elec
D150	sc elec
D173	mc elec (LGN-GL4)
R178	np + sc elec (RGS14-GL) / np (LGN-GL4, LGN-GL3)
V179	np + mc elec
K180	np (RGS14-GL, LGN-GL4) / np + sc elec (LGN-GL3)
T181	sc elec (LGN-GL4) / np (LGN-GL3)
D200	sc elec
G202	np + mc elec (RGS14-GL) / np (LGN-GL4, LGN-GL3)
G203	np
R205	np (RGS14-GL, LGN-GL4)
S206	mc elec (RGS14-GL)
E207	np (LGN-GL3)
R208	np + sc elec (RGS14-GL, LGN-GL4)
W211	np
I212	np (LGN-GL4, LGN-GL3)
F215	np
D229	sc elec (RGS14-GL, LGN-GL4)

E238	sc elec (LGN-GL4) / np + sc elec (LGN-GL3)
R242	np + sc elec (RGS14-GL, LGN-GL3) / np (LGN-GL4)
E245	np (RGS14-GL, LGN-GL3) / sc elec (LGN-GL4)
K248	np
L249	np
S252	np
I253	np (LGN-GL3)
N256	np
W258	np (LGN-GL4, LGN-GL3)
F259	np (LGN-GL3)
D272	sc elec (LGN-GL4, LGN-GL3)

[a] Energy contributions, calculated as described in Methods: np, non-polar; sc elec, side-chain electrostatic contribution; mc elec, main chain electrostatic contribution.
